# Transfer of Neuroplasticity from Nucleus Accumbens Core to Shell Is Required for Cocaine Reward

**DOI:** 10.1371/journal.pone.0030241

**Published:** 2012-01-17

**Authors:** Nicolas Marie, Corinne Canestrelli, Florence Noble

**Affiliations:** 1 Centre National de la Recherche Scientifique, Unité Mixte de Recherche 8206, Paris, France; 2 Institut National de la Santé et de la Recherche Médicale, U 705, Paris, France; 3 Université Paris Descartes, Laboratoire de Neuropsychopharmacologie des Addictions, Paris, France; Hokkaido University, Japan

## Abstract

It is well established that cocaine induces an increase of dendritic spines density in some brain regions. However, few studies have addressed the role of this neuroplastic changes in cocaine rewarding effects and have often led to contradictory results. So, we hypothesized that using a rigorous time- and subject-matched protocol would demonstrate the role of this spine increase in cocaine reward. We designed our experiments such as the same animals (rats) were used for spine analysis and behavioral studies. Cocaine rewarding effects were assessed with the conditioned place preference paradigm. Spines densities were measured in the two subdivisions of the nucleus accumbens (NAcc), core and shell. We showed a correlation between the increase of spine density in NAcc core and shell and cocaine rewarding effects. Interestingly, when cocaine was administered in home cages, spine density was increase in NAcc core only. With anisomycin, a protein synthesis inhibitor, injected in the core we blocked spine increase in core and shell and also cocaine rewarding effects. Strikingly, whereas injection of this inhibitor in the shell immediately after conditioning had no effect on neuroplasticity or behavior, its injection 4 hours after conditioning was able to block neuroplasticity in shell only and cocaine-induced place preference. Thus, it clearly appears that the neuronal plasticity in the NAcc core is essential to induce plasticity in the shell, necessary for cocaine reward. Altogether, our data revealed a new mechanism in the NAcc functioning where a neuroplasticity transfer occurred from core to shell.

## Introduction

Cocaine, like most drugs of abuse, is able to increase dopamine in the nucleus accumbens (NAcc) [1]. This effect is recognized to be at the origin of the reinforcing properties of this psychostimulant [2]. NAcc is part of the rewarding cortico-mesolimbic dopamine pathway. This is a heterogeneous structure divided in two subregions termed core and shell, differentially involved in cocaine rewarding effects. Thus, in the conditioned-place preference (CPP) paradigm, a test assessing animal's ability to associate drug-induced effects with environmental cues to quantify drug reward, intra-shell but not intra-core injection of cocaine induces CPP [3]. Moreover lesion of medial shell by 6-hydroxydopamine inhibits cocaine-induced CPP, whereas core lesion has no effect [4] demonstrating that the NAcc core and shell may have different functions in cocaine rewarding effects.

The vast majority of the NAcc is composed of GABAergic medium spiny neurons (MSN). Thanks to their dendritic spines (DeS), MSN are responsible for integration of dopaminergic and glutamatergic signaling as they receive dopaminergic axons from the ventral tegmental area (VTA) (connected to spine neck) and glutamatergic inputs from prefrontal cortex (connected to spine head) [5]. This integration is of a particular importance since dopamine and glutamate play major roles in neurobiological effects of cocaine [6]. While cocaine has been shown to increase DeS density in the NAcc, its relationship with behavior is far to be clear. Indeed, whereas some authors have shown that cocaine-induced behavioral sensitization or cocaine self-administration alters DeS density in the NAcc [7], [8], others failed to observe a DeS increase after cocaine treatment [9] known to promote behavioral sensitization [10]. These discrepancies might be explained by different cocaine regimens or withdrawal period. Moreover, blocking cocaine-dependent DeS increase seems to have opposite effect on behavior. For instance, in animals expressing a high MEF2 (myocyte-enhancer factor 2) activity, cocaine was unable to induce DeS increase in the NAcc but its rewarding effect was enhanced [11]. Conversely, in animals expressing a dominant negative mutant of NFκB (nuclear factor kappa B) [12] or in kalirin7 knock-out mice [13], cocaine failed to induce DeS increase and was devoid of rewarding effects evidenced by CPP. We hypothesized that these conflicting results have their origin in the different protocols used for DeS analysis and behavior because DeS analysis was not realized on brain from animals enrolled in behavioral experiments.

So, we used a rigorous time- and subject-matched protocol to demonstrate the role of dendritic spine in cocaine reward (measured by CPP). With an age-dependent model of vulnerability to cocaine rewarding effect we found that CPP correlated with DeS increase in NAcc core and shell. Interestingly, when cocaine treatment was administered in home cages (in absence of conditioning), neuroplasticity was only observed in the NAcc core. We finally attempted to determine the role of DeS in behavior by microinjection of anisomycin (a protein synthesis inhibitor) in NAcc core or shell. Whereas anisomycin injection in the core blocked DeS increase in both core and shell and cocaine-induced CPP, anisomycin injection in the shell 4 hours after conditioning prevented DeS increase in the shell and CPP. These results revealed a new mechanism of Nacc functioning where a transfer of neuroplasticity from core to shell was required for cocaine reward.

## Materials and Methods

### Chemicals

Paraformaldehyde (PFA), electron microscopy grade, was from E.M.S (Hatfield, USA). Cocaine hydrochloride, 1,4-Diazabicyclo[2.2.2]octane (DABCO) and anisomycin were from Sigma-Aldrich (Lyon, France). 1,1′-dioctadecyl-3,3,3′,3′-tetramethylindocarbocyanine perchlorate (DiI) and Dulbecco's Phosphate Buffered Saline (PBS) were from Invitrogen (Cergy Pontoise, France). Mowiol was from Calbiochem (Nottingham, UK).

### Animals and treatments

Male Sprague-Dawley rats (Janvier, Le Genest-Saint-Isle, France) were housed in a temperature- (22±1°C) and humidity-controlled (50±5%) environment and had access to food and water ad libitum. PND0 (Post natal day 0) was the day of birth. Two different ages of rats were used : adolescent (PND27 at the first cocaine injection) and adult (PND70 at the first cocaine injection). Experiments were carried out in accordance with the European Communities Council Directive of 24 November 1986 (86/609/EEC) for the care of laboratory animals. All animal care and experimental procedures were approved by the local ethics committee of the faculty of pharmacy (Université Paris Descartes), approval ID P2-FN-152-10.

Cocaine was dissolved in saline (0.9% NaCl) and animals received 1 ml/kg of body weight via intraperitoneal (i.p.) route.

### Conditioned place preference (CPP) procedure

We used an unbiased CPP procedure. The place preference apparatus consisted of two conditioning compartments (45×45×30 cm) separated by a neutral compartment (18×36×30 cm). The conditioning compartments had distinct sensory clues : black walls with rough floor, and white stripes walls with smooth floor. The neutral compartment had gray walls and floor. In all experiments, rats did not show any preference for the black or striped compartment during the preconditioning phase. The drug-associated compartment was randomized across subjects and treatments in order to normalize small biases that might occur. The movement and location of animals were recorded by computerized monitoring software (Videotrack, Viewpoint, Lyon, France). The protocol consisted of three phases: 1) Preconditioning phase (1 day): drug-naive animals had free access to both compartments for 20 min, and the time spent in each compartment was recorded. 2) Conditioning phase: this phase consisted of 4 days in which each conditioning chamber was closed. In the morning of the first conditioning day, animals were treated with saline and placed in one of the conditioning environments individually for 20 min. In the afternoon, the animals were given cocaine in the opposite compartment and this sequence alternated during the next 3 days. Control animals received saline twice a day and were submitted to an alternated sequence between the two compartments. 3) Test phase (1 day): This phase took place 24 h after the final conditioning session and was carried out exactly as the preconditioning phase. Results were expressed in score (in s) calculated as the difference between the time spent in the drug-paired compartment during the postconditioning phase minus the time spent in the same compartment during the test phase.

### Stereotaxic surgery

Rats were anesthetized with ketamine (80 mg/kg i.p.)/xylazine (10 mg/kg, i.p.) and placed in a stereotaxic frame (David Kopf Instruments, David Kopf Instruments, Tujunga, USA). The skull was exposed and a guide cannula (length 13 mm) was bilaterally implanted in the NAcc core or shell. The coordinates, taken from the atlas of Paxinos and Watson [14] were +1.7 mm from the bregma, ±1,8 mm lateral to the midline, and −3.3 mm under the skull surface for the core and +1.2 mm from the bregma, ±0.8 mm lateral to the midline, and −3.7 mm under the skull surface for the shell. Animals were used for experiments after a recovery period of 5 days.

### Anisomycin treatment during CPP acquisition

Anisomycin was dissolved in 1 N HCl, diluted in artificial cerebrospinal fluid (140 mM NaCl, 4 mM KCl, 1.2 mM CaCl_2_, 1 mM MgCl_2_, 1.9 mM Na_2_HPO_4_, 0.1 mM NaH_2_PO_4_, pH 7.4) and pH was adjusted to 7.4 (final concentration was 100 µg/µl). After each afternoon conditioning session, a removable needle (30 G×16 mm) was inserted into the guide cannula. Needle was connected to a glass Hamilton syringe via polyethylene tubing. Syringe was driven by a microinfusion pump and a volume of 0.5 µl/side was injected at a flow rate of 0.4 µl/min. This corresponds to 50 µg/side of anisomycin, a quantity demonstrated to block protein synthesis [15]. Cannula track was verified visually on slices used for dendritic spine staining (see below). Animals with cannula placements outside of the Nacc core or shell subregions were excluded from subsequent data analysis.

### Visualization of dendritic spines

This protocol is based on a light fixation of brain with PFA to preserve dendritic spines structure [16].

#### Preparation of fixed brain slices

Rats were deeply anesthetized by a i.p. injection of sodium pentobarbital (100 mg/kg) and brains were fixed with intracardiac perfusion of freshly prepared ice-cold 1.5% PFA in 0.1 M phosphate buffer (PB) for 15 min at 20 ml/min using a peristaltic pump. Brains were dissected and post-fixed in 1.5% PFA/0.1 M PB for 1 h at 4°C then transferred to PBS. After 2 washes in PBS, brain coronal sections containing the nucleus accumbens were collected in PBS by sectioning the brain into 100 µm slices using a vibratome (Leica VT 1000E).

#### Dendritic spine staining

Solid DiI crystals were applied on the surface of the slice using an acupuncture needle. Slices were left at room temperature (RT) for 6 hours to allow dye diffusion along the neuronal membrane, carefully washed once with PBS and fixed again in 4% PFA/PBS for 30 min at RT. Slices were finally mounted in a glycerol-based mounting medium Mowiol to avoid possible dehydration-induced shrinkage of dendritic structures [17] containing DABCO as an antifade reagent.

#### Confocal imaging and quantification of dendritic spines

**Figure 1:**
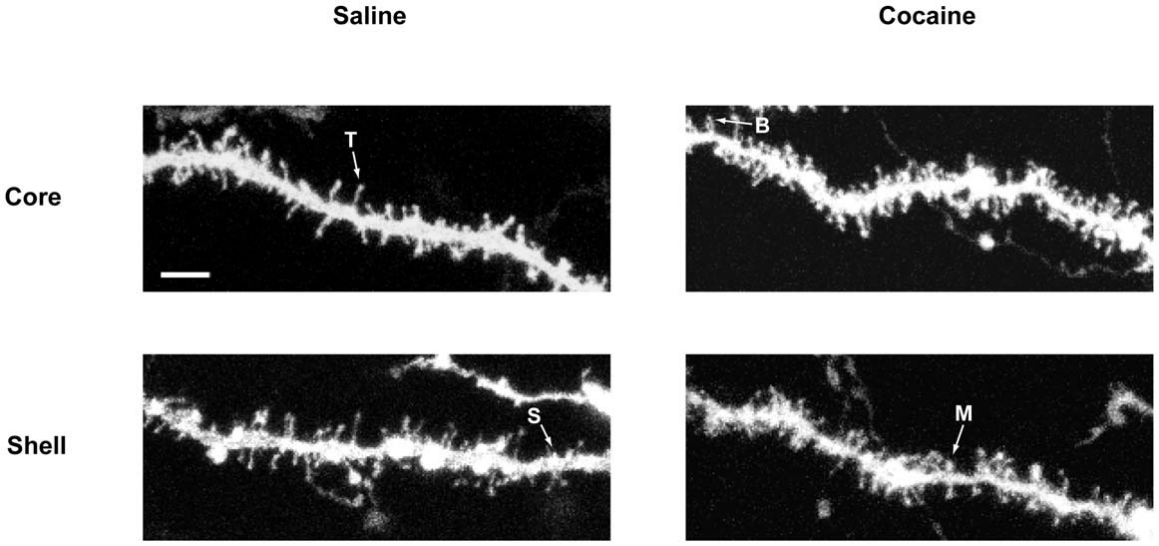
Representative dendritic segment of medium spiny neurons from NAcc core or Shell. Images were obtained from adult rats treated with cocaine at 20 mg/kg for CPP induction and showed increase in DeS density in both core and shell after cocaine administration. Brains were removed, fixed, and DeS were visualized using confocal microscope as described in material and methods (scale bar, 5 µm). Only contrast was slightly modified. Arrows indicate thin (T), stubby (S), branched (B) and mushroom (M) spines.

Dendritic spines on medium spiny neurons (MSN) in the NAcc core and shell were imaged using a Leica SP2 confocal laser scanning microscope (Fig. 1). All images were taken using the Plan-APOCHROMAT ×40 oil-immersion lens (N/A 1.4). We used 2048×2048 pixels for frame size without zooming and the fluorescence of DiI was visualized with the 543 nm Helium/Neon laser. Serial stack images with step size ranging from 0.4 to 0.6 µm were collected, and then projected to reconstruct a three dimensional (3D) image using NIH ImageJ software (http://rsbweb.nih.gov/ij/). Protrusions from dendrites are classified in several classes according to their shape : mushroom (large head and short neck), thin (thin head and long neck), stubby (large head and doesn't seem to have a neck), branched (more than one head) and filopodia (lacking discernible head) (Fig. 1) [18], [19], [20], [21]. Except filopodia that will not always give a spine [22], all dendritic protrusion were included in the analysis. Dendritic spines were analyzed on 2^nd^ order dendrites on a length >50 µm [23]. The 3D reconstruction was used to accurately follow a dendrite segment and to clearly identify individual spines. Two dendrites/neuron and 4 to 5 neurons for core or shell were used for counting. So, a density expressed in spines/µm per animal was generated and used for statistical analysis.

All images sets were coded and all measurements were performed by an experimenter blind to the experimental conditions.

### Statistical analysis

All data were expressed as the mean ± S.E.M. and analyzed by comparing the means using one-way ANOVA followed by Dunnett's test (for dose-response experiments), two-way ANOVA followed by a Bonferroni's post-hoc test for multiple comparisons (for anisomycin experiments) or t test (two groups). The relationship between behavioral measures (CPP score) and spine density was analyzed by a Person's correlation test.

## Results

### Vulnerability to cocaine rewarding effects correlates with neuroplasticity in the NAcc

**Figure 2:**
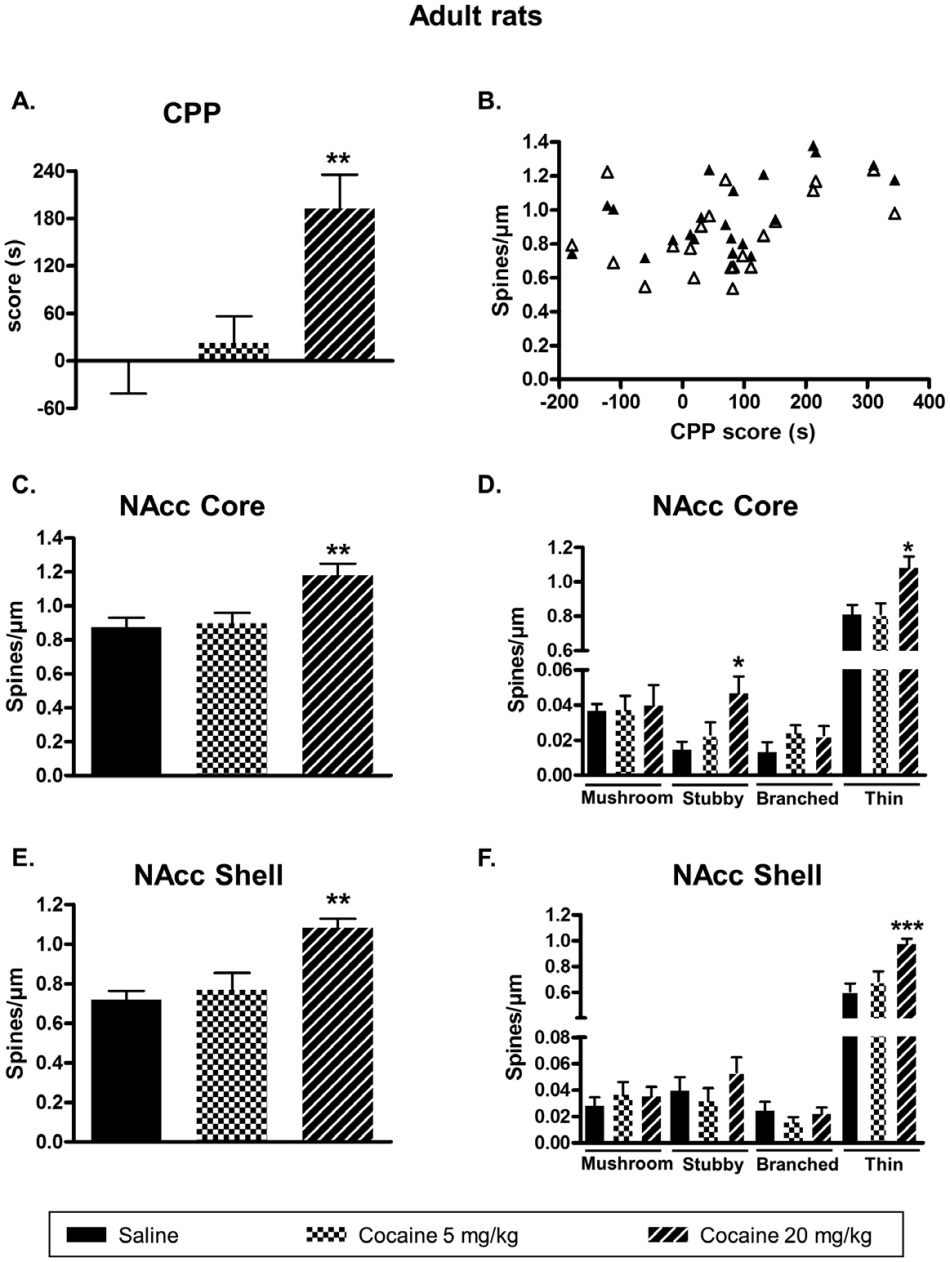
High but not low dose of cocaine induces CPP and DeS increase in the NAcc core and shell of adult rats. CPP was induced in adult rats with cocaine at 5 or 20 mg/kg. Immediately after the test, brains were processed for DeS analysis according to materials and methods. CPP scores were expressed the means ± S.E.M. (A) (one-way ANOVA, F_2,18_ = 7.09, p<0.01). Total DeS density was expressed as spines/µm (mean ± S.E.M.) in MSN from the NAcc core (C) (one-way ANOVA, F_2,18_ = 7.226, p<0.01) and shell (E) (one-way ANOVA, F_2,18_ = 10.21, p<0.01). Pearson correlation analysis between CPP score and DeS density in both core (r = 0.57, p = 0.003, closed triangles) and shell (r = 0.38, p = 0.043, open triangles) is shown in B. Density of mushroom, stubby, branched or thin spines was expressed as spines/µm (mean ± S.E.M.) in MSN from the NAcc core (D) (one-way ANOVA mushroom, F_2,18_ = 0.039, p = 0.961; one-way ANOVA stubby, F_2,18_ = 4.91, p<0.05; one-way ANOVA branched, F_2,18_ = 1.273, p = 0.303; one-way ANOVA thin, F_2,18_ = 6.285, p<0.01) and shell (F) (one-way ANOVA mushroom, F_2,18_ = 0.307, p = 0.738; one-way ANOVA stubby, F_2,18_ = 1.168, p = 0.333; one-way ANOVA branched, F_2,18_ = 0.754, p = 0.484; one-way ANOVA thin, F_2,18_ = 9.915, p<0.01). * p<0.05, ** p<0.01, *** p<0.001 vs saline group, Dunett's test, n = 7 animals/group.

**Figure 3:**
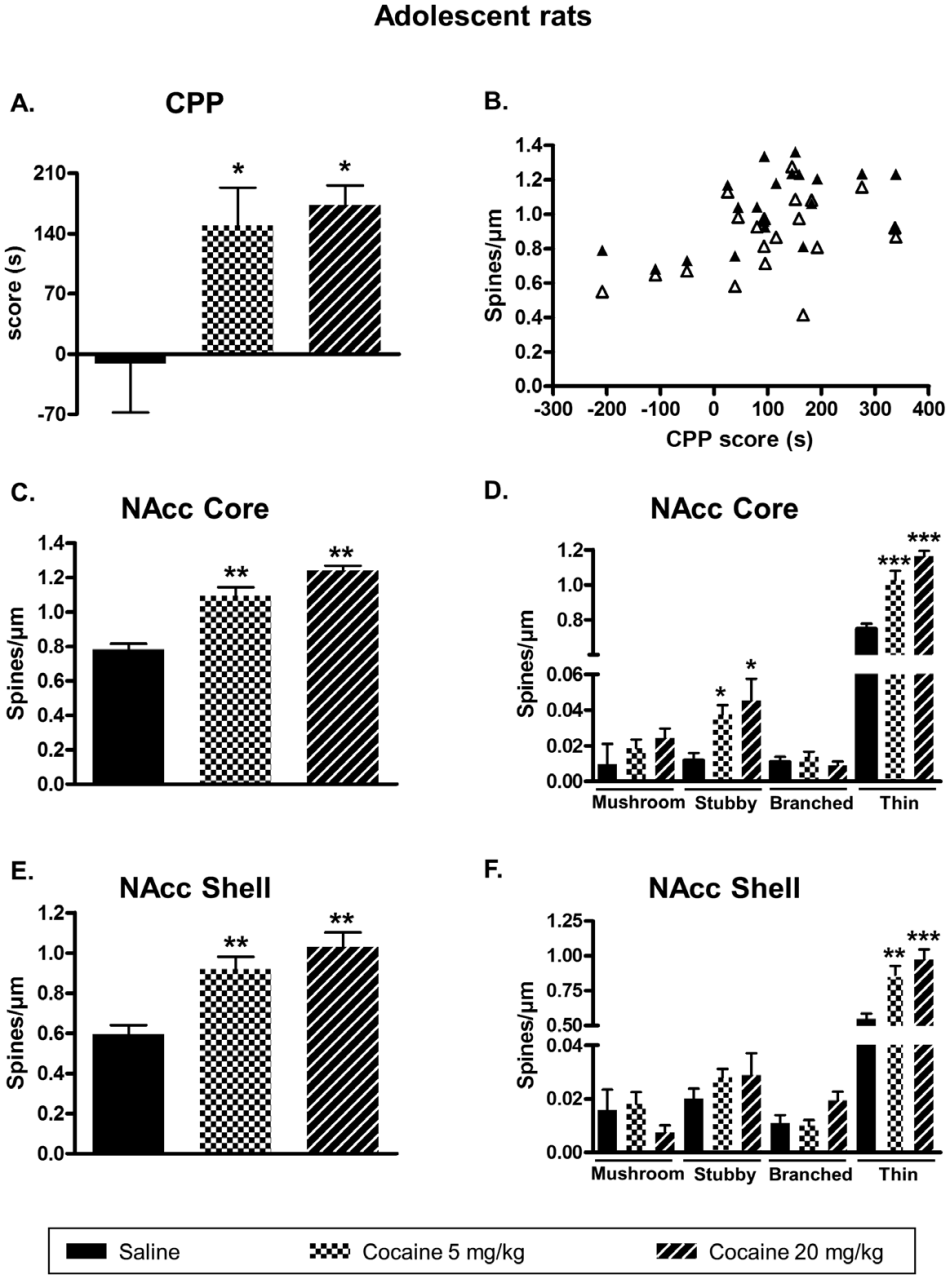
High and low doses of cocaine induce CPP and DeS increase in the NAcc core and shell of adolescent rats. CPP was induced in adolescent rats with cocaine at 5 or 20 mg/kg. Immediately after the test, brains were processed for DeS analysis according to materials and methods. CPP scores were expressed the means ± S.E.M. (A) (one-way ANOVA, F_2,17_ = 4.8, p<0.05). Total DeS density was expressed as spines/µm (mean ± S.E.M.) in MSN from the NAcc core (C) (one-way ANOVA, F_2,17_ = 28.83, p<0.001) and shell (E) (one-way ANOVA, F_2,17_ = 12.25, p<0.001). Pearson correlation analysis between CPP score and DeS density in both core (r = 0.54, p = 0.006, closed triangles) and shell (r = 0.42, p = 0.003, open triangles) is shown in B. Density of mushroom, stubby, branched or thin spines was expressed as spines/µm (mean ± S.E.M.) in MSN from the NAcc core (D) (one-way ANOVA mushroom, F_2,17_ = 1.88, p = 0.181; one-way ANOVA stubby, F_2,17_ = 4.937, p<0.05; one-way ANOVA branched, F_2,17_ = 0.7727, p = 0.477; one-way ANOVA thin, F_2,17_ = 22.36, p<0.001) and shell (F) (one-way ANOVA mushroom, F_2,17_ = 1.143, p = 0.342; one-way ANOVA stubby, F_2,17_ = 0.080, p = 0.461; one-way ANOVA branched, F_2,17_ = 3.326, p = 0.06; one-way ANOVA thin, F_2,17_ = 12.22, p<0.001). * p<0.05, ** p<0.01, *** p<0.001 vs saline group, Dunett's test, n = 6 to 8 animals/group.

To establish a link between behavioral effects and changes in plasticity induced by cocaine, we used a model of age-dependent vulnerability to rewarding effect of cocaine. It is well known that young rats differ from adult animals in cocaine-induced CPP [24]. In this model, we hypothesized that cocaine would promote an increase of DeS in NAcc only when CPP occurred, regardless of the age of the animals or the cocaine doses. In a first set of experiments we measured the ability of two doses of cocaine (5 and 20 mg/kg i.p.) to induce reinforcing effects as measured by CPP in adult and adolescent rats, followed by DeS analysis in the NAcc core and shell. Cocaine treatment and duration of conditioning (4 days) were selected according to previous works [24](see Materials and Methods section). As shown in figure 2A, adult rats demonstrated CPP only at 20 mg/kg cocaine. In adolescent rats, 5 and 20 mg/kg cocaine were able to induce CPP (Fig. 3A).

Immediately after the test in the CPP paradigm, animals were perfused with fixative, their brains removed and fixed for DeS analysis. Spine densities were determined on MSN from core and shell subdivisions of the NAcc as these structures play a role in drug reward [25]. In saline-treated animals, we found spine densities in the NAcc core of 0.87±0.05 and 0.78±0.03 spine/µm for adult (Fig. 2C) and adolescent rats (Fig. 3C), respectively. This was slightly lower to other findings where spine density was around 1 spine/µm for the NAcc core [23], [26]. This discrepancy could be explained by our choice of excluding filopodia from spine counting as these structures will not always lead to spines [22]. In the NAcc shell, spine density is generally found to be lower than in the core [23], [26]. Accordingly, in the NAcc shell from saline-treated animals spine densities were 0.71±0.04 and 0.59±0.04 spine/µm for adults (Fig. 2E) and adolescent rats (Fig. 3E), respectively. In drug-treated animals, adult rats demonstrated an increase of DeS in both core (Fig. 2C) and shell (Fig. 2E) only for the dose of cocaine that produced CPP (Fig. 2A). On the other hand, in adolescent rats where CPP was obtained with the two cocaine regimens (5 or 20 mg/kg), both these doses increased DeS density in the NAcc core (Fig. 3C) and shell (Fig. 3E). A detailed analysis of spine type demonstrated that stubby and thin spines were increased after cocaine-induced CPP in NAcc core (Fig. 2D and 3D) whereas only thin spines were augmented in NAcc shell (Fig. 2F and 3F). Subsequent statistical analysis revealed that CPP score positively correlates with dendritic spine density for both adult (Fig. 2B) and adolescent rats (Fig. 3B).

### Cocaine treatment in home cages induces neuroplasticity in the NAcc core only

**Figure 4:**
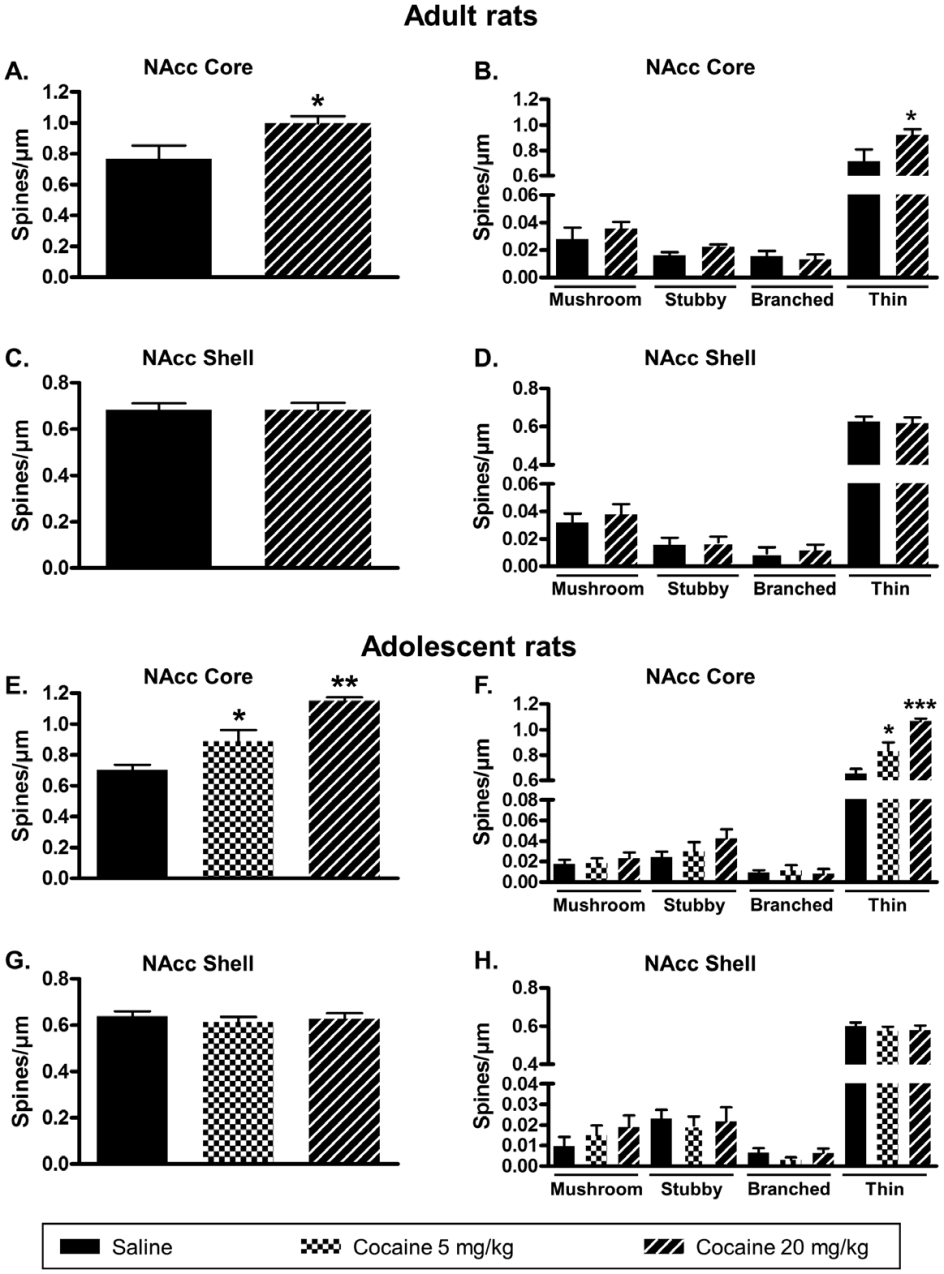
Cocaine treatments in home cages, at doses efficient to induce CPP, promote DeS increase only in the NAcc core. Adult (A, B, C, D) or adolescent (E, F, G, H) rats received cocaine at 5 or 20 mg/kg for 4 days in the same conditions as CPP but animals were placed in their home cage after each injection. 24 hours after the last injection, brains were processed for DeS analysis in MSN from the NAcc core (A, B, E, F) and shell (C, D, G, H). Density of total (A, C, E, G) or different types (B, D, F, H) of spine was expressed as spines/µm (mean ± S.E.M.). One-way ANOVA, F_2,25_ = 24.42, p<0.001 (E); F_2,25_ = 0.3025, p = 0.741 (G). One-way ANOVA mushroom, F_2,25_ = 0.37, p = 0.694; one-way ANOVA stubby, F_2,25_ = 1.368, p = 0.273; one-way ANOVA branched, F_2,25_ = 0.1781, p = 0.837; one-way ANOVA thin, F_2,25_ = 21.9, p<0.001 (F). One-way ANOVA mushroom, F_2,25_ = 0.817, p = 0.453; one-way ANOVA stubby, F_2,25_ = 0.081, p = 0.9223; one-way ANOVA branched, F_2,25_ = 1.039, p = 0.368; one-way ANOVA thin, F_2,25_ = 0.328, p = 0.723 (H). * p<0.05, ** p<0.01, *** p<0.001 vs saline group, Dunnett's test, n = 9 to 10 animals/group (E, F, G, H). * p<0.05, t test, n = 7 to 10 animals/group (A, B, C, D).

In a second set of experiments, we determined if cocaine treatment in adult and adolescent rats in home cages, at doses efficient for inducing CPP, involved different patterns in the NAcc neuroplasticity [8]. Animals were treated with the same scheme as in the CPP paradigm (4 days treatment with saline in the morning, and saline or cocaine in the afternoon) except they were placed in their home cages after each injection. 24 hours after the last injection, animals were perfused with fixative, their brains removed and fixed for DeS analysis. When adults rats were treated with 20 mg/kg cocaine a significant increase of DeS density was observed in the NAcc core (Fig. 4A) but not the shell (Fig. 4C). In adolescent rats, a significant increase of DeS density was observed in the NAcc core at both 5 and 20 mg/kg cocaine (Fig. 4E) whereas no variations were detected in the shell (Fig. 4G). Interestingly, only thin spines were increased in NAcc core after cocaine treatment (Fig. 4B and 4F) whereas no changes in the four types of spines were measured in the shell (Fig. 4D and 4H).

### Effects of anisomycin on behavior and neuroplasticity induced by cocaine

**Figure 5:**
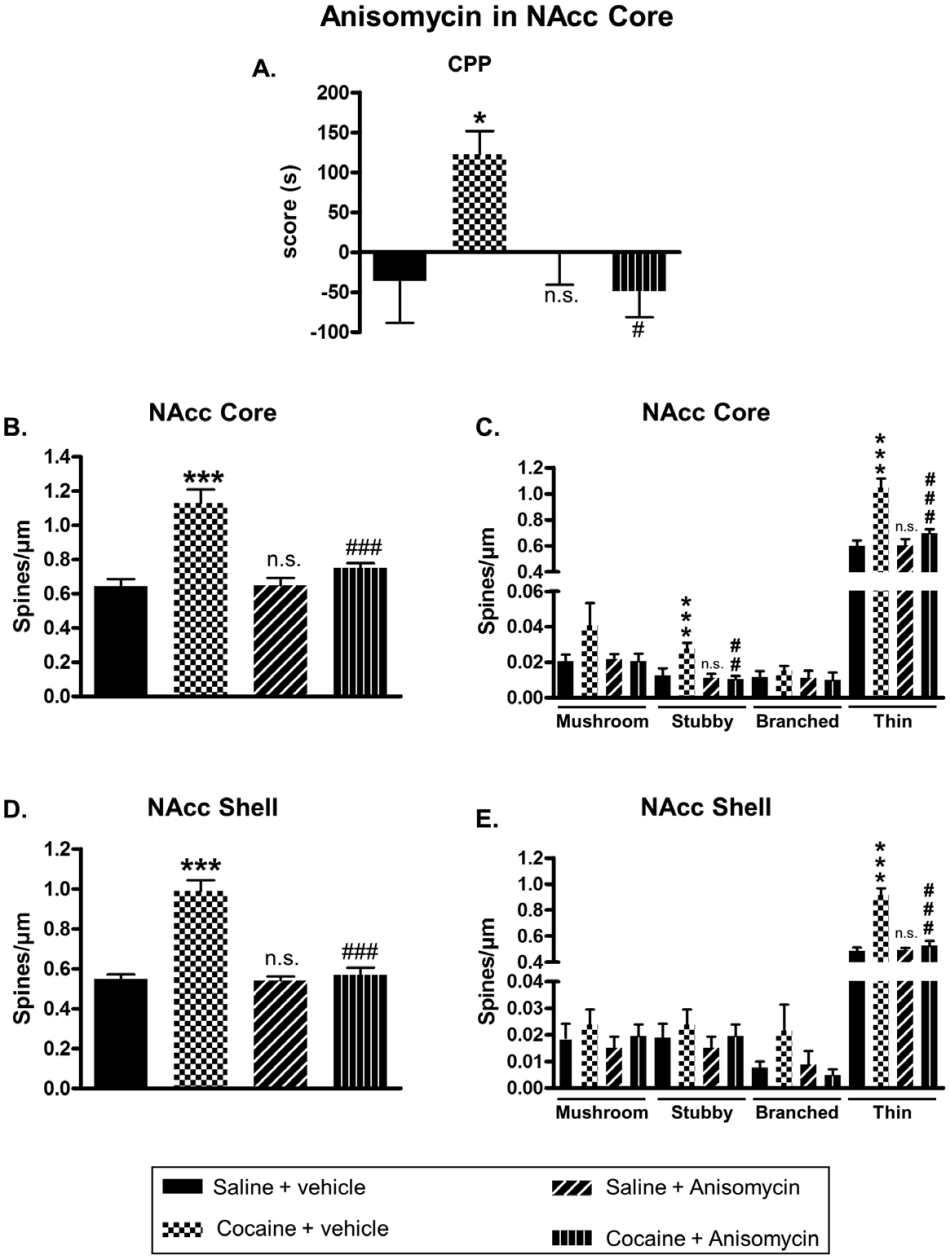
Anisomycin injection in the NAcc core blocks cocaine-induced CPP and neuroplasticity in the NAcc core and shell. CPP was induced in adult rats with saline or cocaine at 20 mg/kg. After each afternoon conditioning session, rats received intra-core infusion of anisomycin or vehicle. Immediately after the test, brains were processed for DeS analysis. CPP scores were expressed as the means ± S.E.M. (A) (Two-way ANOVA, F_cocaine(1,30)_ = 6.364, p = 0.18; F_anisomycin(1,30)_ = 2.851, p = 0.101; F_cocaine × anisomycin_ = 6.364, p<0.05). Total DeS density was expressed as spines/µm (mean ± S.E.M.) in MSN from the NAcc core (B) (Two-way ANOVA, F_cocaine(1,30)_ = 36.03, p<0.001; F_anisomycin(1,30)_ = 14.86, p<0.001; F_cocaine × anisomycin_ = 15.51, p<0.001) and shell (D) (Two- way ANOVA, F_cocaine(1,30)_ = 48.46, p<0.001; F_anisomycin(1,30)_ = 39.31, p<0.001; F_cocaine × anisomycin_ = 37.72, p<0.001). Density of mushroom, stubby, branched or thin spines was expressed as spines/µm (mean ± S.E.M.) in MSN from the NAcc core (C) (Two-way ANOVA mushroom, F_cocaine(1,30)_ = 2.636, p = 0.114; F_anisomycin(1,30)_ = 2.096, p = 0.158; F_cocaine × anisomycin_ = 3.405, p = 0.074; Two-way ANOVA stubby, F_cocaine(1,30)_ = 5.852, p<0.05; F_anisomycin(1,30)_ = 9.828, p<0.05; F_cocaine × anisomycin_ = 6.994, p<0.05; Two-way ANOVA branched, F_cocaine(1,30)_ = 0.081, p = 0.776; F_anisomycin(1,30)_ = 0.626, p = 0.434; F_cocaine × anisomycin_ = 0.419, p = 0.522; Two-way ANOVA thin, F_cocaine(1,30)_ = 33.43, p<0.001; F_anisomycin(1,30)_ = 13.75, p<0.001; F_cocaine × anisomycin_ = 14.33, p<0.001) and shell (E) (Two-way ANOVA mushroom, F_cocaine(1,30)_ = 0.574, p = 0.454; F_anisomycin(1,30)_ = 2.215, p = 0.147; F_cocaine × anisomycin_ = 0.03, p = 0.861; Two-way ANOVA stubby, F_cocaine(1,30)_ = 0.944, p = 0.338; F_anisomycin(1,30)_ = 0.62, p = 0.426; F_cocaine × anisomycin_ = 0.004, p = 0.949; Two-way ANOVA branched, F_cocaine(1,30)_ = 0.942, p = 0.339; F_anisomycin(1,30)_ = 2.319, p = 0.138; F_cocaine × anisomycin_ = 3.028, p = 0.092; Two-way ANOVA thin, F_cocaine(1,30)_ = 44.43, p<0.001; F_anisomycin(1,30)_ = 30.71, p<0.001; F_cocaine × anisomycin_ = 32.67, p<0.001). * p<0.05; *** p<0.001; n.s. (not significant) vs Saline + vehicle group; # p<0.05; ### p<0.001 vs Cocaine + vehicle group, Bonferroni post-hoc test, n = 7 to 9 animals/group.

**Figure 6:**
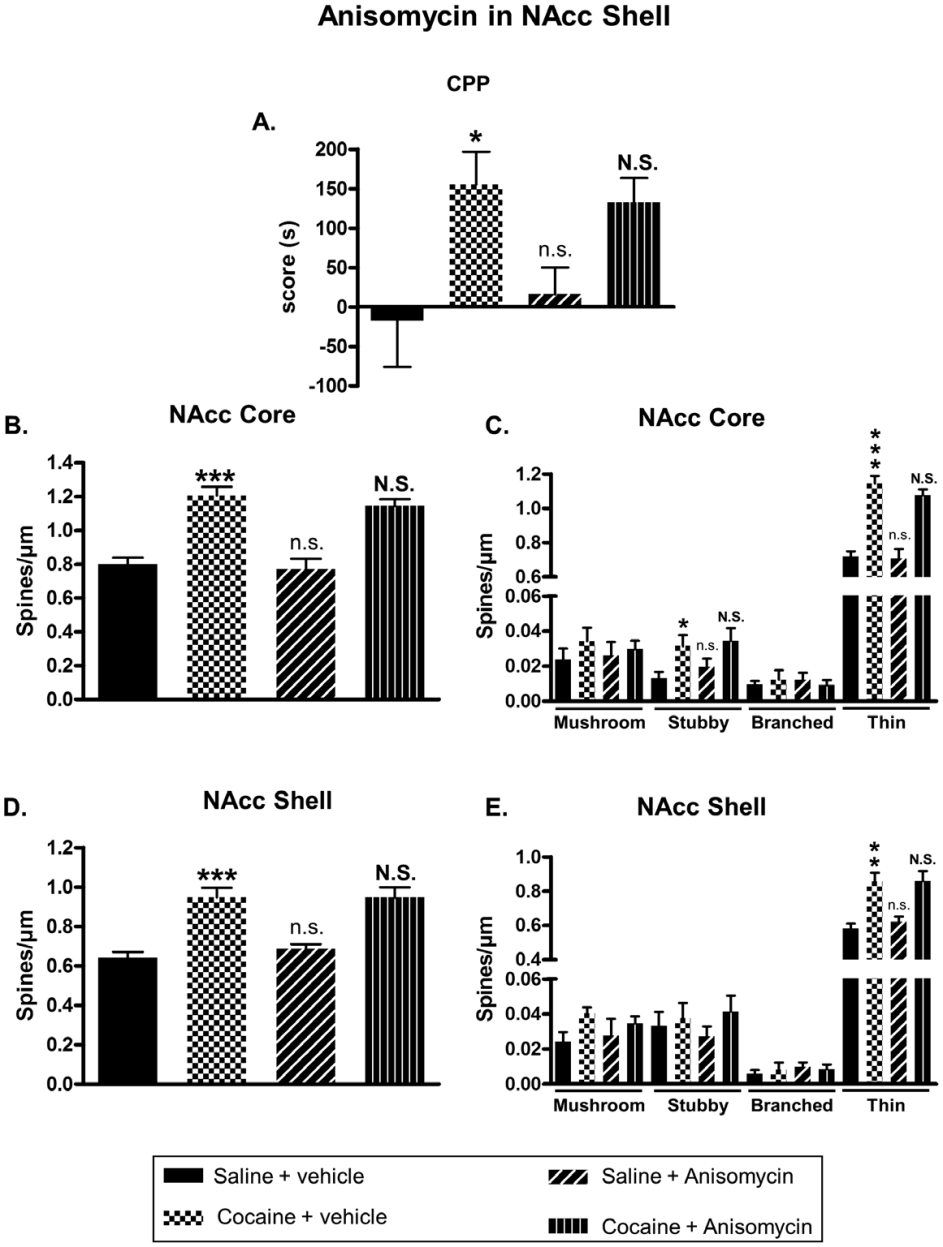
Anisomycin injection in the shell immediately after cocaine conditioning is devoid of any effects on CPP and neuroplasticity in the NAcc core and shell. CPP was induced in adult rats with saline or cocaine at 20 mg/kg. After each afternoon conditioning session, rats received intra-shell infusion of anisomycin or vehicle. Immediately after the test, brains were processed for DeS analysis. CPP scores were expressed as the means ± S.E.M. (A) (Two-way ANOVA, F_cocaine(1,32)_ = 10.68, p<0.01; F_anisomycin(1,32)_ = 0.01307, p = 0.909; F_cocaine × anisomycin_ = 0.4087, p = 0.527). Total DeS density was expressed as spines/µm (mean ± S.E.M.) in MSN from the NAcc core (B) (Two-way ANOVA, F_cocaine(1,32)_ = 61.75, p<0.001; F_anisomycin(1,32)_ = 0.8887, p = 0.352; F_cocaine × anisomycin_ = 0.1014, p = 0.752) and shell (D) (Two-way ANOVA, F_cocaine(1,32)_ = 52.96, p<0.001; F_anisomycin(1,32)_ = 0.3396, p = 0.564; F_cocaine × anisomycin_ = 0.3232, p = 0.573). Density of mushroom, stubby, branched or thin spines was expressed as spines/µm (mean ± S.E.M.) in MSN from the NAcc core (C) (Two-way ANOVA mushroom, F_cocaine(1,32)_ = 1.098, p = 0.302; F_anisomycin(1,32)_ = 0.024, p = 0.877; F_cocaine × anisomycin_ = 0.247, p = 0.621; Two-way ANOVA stubby, F_cocaine(1,32)_ = 8.046, p<0.01; F_anisomycin(1,32)_ = 0.646, p = 0.427; F_cocaine × anisomycin_ = 0.08, p = 0.778; Two-way ANOVA branched, F_cocaine(1,32)_ = 0.005, p = 0.942; F_anisomycin(1,32)_ = 0.005, p = 0.939; F_cocaine × anisomycin_ = 0.666, p = 0.42; Two-way ANOVA thin, F_cocaine(1,32)_ = 82.94, p<0.001; F_anisomycin(1,32)_ = 0.779, p = 0.384; F_cocaine × anisomycin_ = 0.369, p = 0.547) and shell (E) (Two-way ANOVA mushroom, F_cocaine(1,32)_ = 3.143, p = 0.085; F_anisomycin(1,32)_ = 0.03, p = 0.862; F_cocaine × anisomycin_ = 0.484, p = 0.491; Two-way ANOVA stubby, F_cocaine(1,32)_ = 1.353, p = 0.253; F_anisomycin(1,32)_ = 0.019, p = 0.891; F_cocaine × anisomycin_ = 0.382, p = 0.54; Two-way ANOVA branched, F_cocaine(1,32)_ = 0.04, p = 0.841; F_anisomycin(1,32)_ = 0.544, p = 0.465; F_cocaine × anisomycin_ = 0.419, p = 0.521; Two-way ANOVA thin, F_cocaine(1,32)_ = 34.31, p<0.001; F_anisomycin(1,32)_ = 0.233, p = 0.632; F_cocaine × anisomycin_ = 0.206, p = 0.653).* p<0.05; ** p<0.01; *** p<0.001; n.s. (not significant) vs Saline + veh group. **N.S.** (not significant) vs Coc + veh, Bonferroni post-hoc test, n = 7 to 10 animals/group.

**Figure 7:**
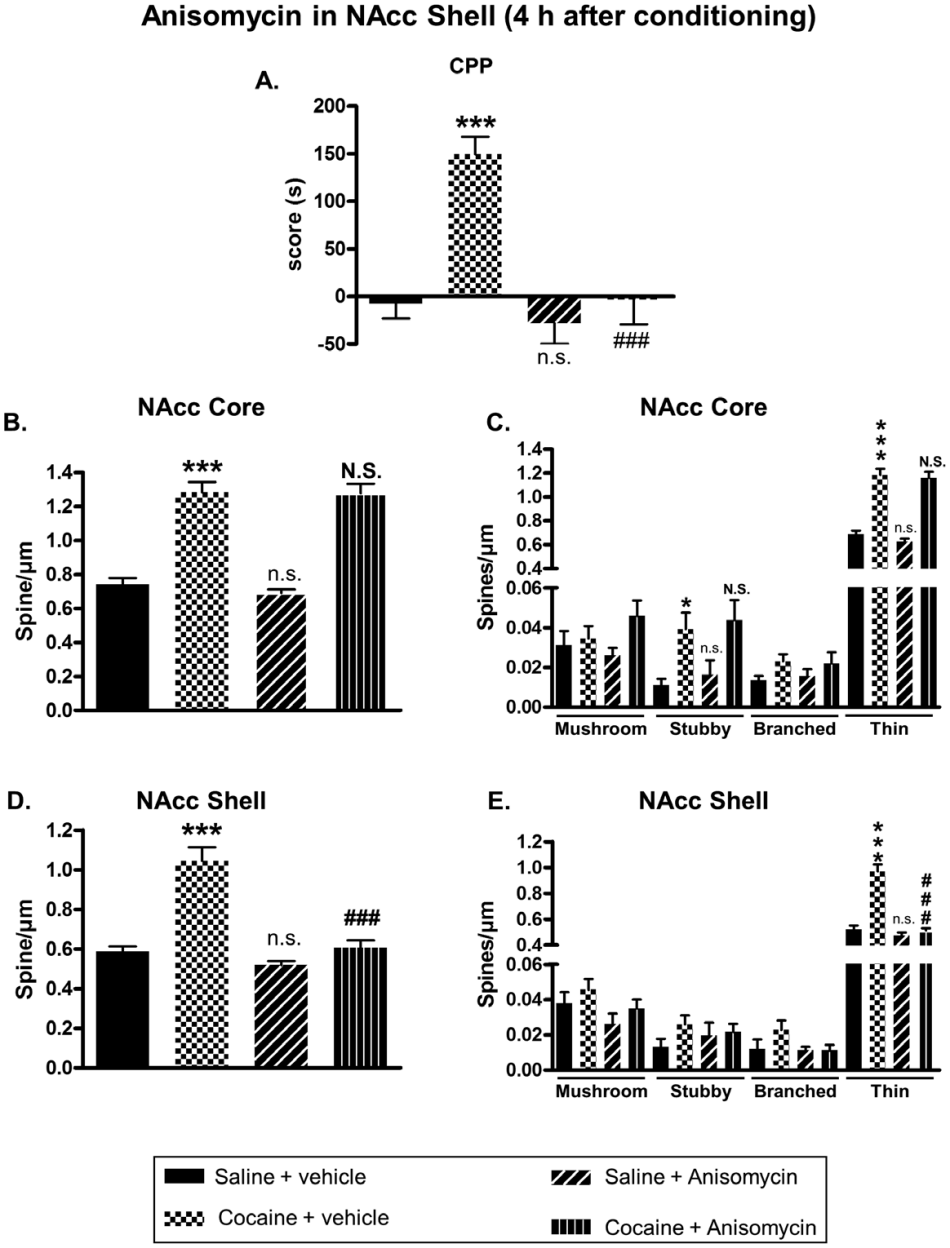
Anisomycin injection in the shell 4 hours after cocaine conditioning blocks cocaine-induced CPP and neuroplasticity in the NAcc shell. CPP was induced in adult rats with saline or cocaine at 20 mg/kg. Four hours after each afternoon conditioning session, rats received intra-shell infusion of anisomycin or vehicle. Just after the test, brains were processed for DeS analysis. CPP scores were expressed as the means ± S.E.M. (A) (Two-way ANOVA, F_cocaine(1,31)_ = 19.55, p<0.001; F_anisomycin(1,31)_ = 17.75 , p<0.001; F_cocaine × anisomycin_ = 10.39, p<0.01). Total DeS density was expressed as spines/µm (mean ± S.E.M.) in MSN from the NAcc core (B) (Two-way ANOVA, F_cocaine(1,31)_ = 147.6, p<0.001; F_anisomycin(1,31)_ = 0.4409, p = 0.511 ; F_cocaine × anisomycin_ = 0.2943, p = 0.591) and shell (D) (Two-way ANOVA, F_cocaine(1,31)_ = 45.27, p<0.001; F_anisomycin(1,31)_ = 38.87, p<0.001; F_cocaine × anisomycin_ = 21, p<0.001). Density of mushroom, stubby, branched or thin spines was expressed as spines/µm (mean ± S.E.M.) in MSN from the NAcc core (C) (Two-way ANOVA mushroom, F_cocaine(1,32)_ = 3.326, p = 0.077; F_anisomycin(1,31)_ = 0.218, p = 0.643; F_cocaine × anisomycin_ = 1.722, p = 0.199; Two-way ANOVA stubby, F_cocaine(1,31)_ = 14.07, p<0.001; F_anisomycin(1,31)_ = 0.421, p = 0.52; F_cocaine × anisomycin_ = 0.001, p = 0.966; Two-way ANOVA branched, F_cocaine(1,31)_ = 4.068, p = 0.052; F_anisomycin(1,31)_ = 0.023, p = 0.879; F_cocaine × anisomycin_ = 0.169, p = 0.683; Two-way ANOVA thin, F_cocaine(1,31)_ = 154.2, p<0.001; F_anisomycin(1,31)_ = 1.127, p = 0.296; F_cocaine × anisomycin_ = 0.164, p = 0.687) and shell (E) (Two-way ANOVA mushroom, F_cocaine(1,32)_ = 1.803, p = 0.189; F_anisomycin(1,31)_ = 3.207, p = 0.083; F_cocaine × anisomycin_ = 0.008, p = 0.926; Two-way ANOVA stubby, F_cocaine(1,31)_ = 1.902, p = 0.177; F_anisomycin(1,31)_ = 0.036, p = 0.849; F_cocaine × anisomycin_ = 0.964, p = 0.336; Two-way ANOVA branched, F_cocaine(1,31)_ = 1.584, p = 0.217; F_anisomycin(1,31)_ = 2.03, p = 0.164; F_cocaine × anisomycin_ = 1.592, p = 0.216; Two-way ANOVA thin, F_cocaine(1,31)_ = 56.29, p<0.001; F_anisomycin(1,31)_ = 60.74, p<0.001; F_cocaine × anisomycin_ = 38.69, p<0.001). * p<0.05; *** p<0.001; n.s. (not significant) vs Saline + vehicle group; ## p<0.01; ### p<0.001; **N.S.** (not significant) vs Cocaine + vehicle group, Bonferroni post-hoc test, n = 8 to 10 animals/group.

**Figure 8:**
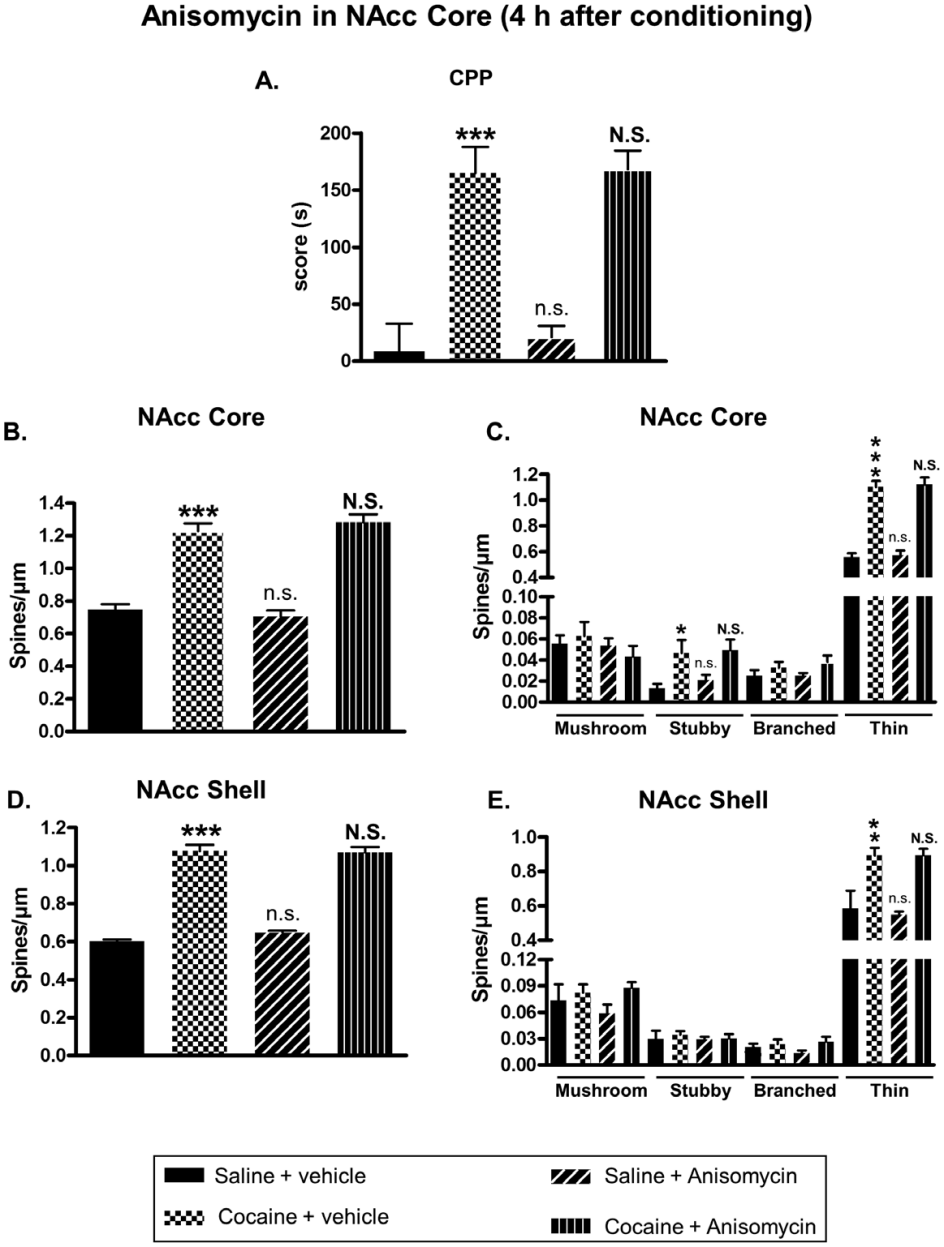
Anisomycin injection in the core 4 hours after cocaine conditioning has no effect on cocaine-induced CPP or neuroplasticity in the NAcc core and shell. CPP was induced in adult rats with saline or cocaine at 20 mg/kg. Four hours after each afternoon conditioning session, rats received intra-core infusion of anisomycin or vehicle. Just after the test, brains were processed for DeS analysis. CPP scores were expressed as the means ± S.E.M. (A) (Two-way ANOVA, F_cocaine(1,31)_ = 60.75, p<0.001; F_anisomycin(1,31)_ = 0.162 , p = 0.69; F_cocaine × anisomycin_ = 0.0548, p = 0.816). Total DeS density was expressed as spines/µm (mean ± S.E.M.) in MSN from the NAcc core (B) (Two-way ANOVA, F_cocaine(1,31)_ = 142.1, p<0.001; F_anisomycin(1,31)_ = 0.0197, p = 0.889; F_cocaine × anisomycin_ = 1.216, p = 0.278) and shell (D) (Two-way ANOVA, F_cocaine(1,31)_ = 327.7, p<0.001; F_anisomycin(1,31)_ = 0.6086, p = 0.441; F_cocaine × anisomycin_ = 1.117, p = 0.298). Density of mushroom, stubby, branched or thin spines was expressed as spines/µm (mean ± S.E.M.) in MSN from the NAcc core (C) (Two-way ANOVA mushroom, F_cocaine(1,32)_ = 0.0189, p = 0.891; F_anisomycin(1,31)_ = 1.2, p = 0.281; F_cocaine × anisomycin_ = 0.7711, p = 0.386; Two-way ANOVA stubby, F_cocaine(1,31)_ = 12.01, p<0.01; F_anisomycin(1,31)_ = 0.3576, p = 0.554; F_cocaine × anisomycin_ = 0.0765, p = 0.783; Two-way ANOVA branched, F_cocaine(1,31)_ = 4.099, p = 0.051; F_anisomycin(1,31)_ = 0.311, p = 0.58; F_cocaine × anisomycin_ = 0.311, p = 0.58; Two-way ANOVA thin, F_cocaine(1,31)_ = 154.3, p<0.001; F_anisomycin(1,31)_ = 0.126, p = 0.724; F_cocaine × anisomycin_ = 0.005, p = 0.938) and shell (E) (Two-way ANOVA mushroom, F_cocaine(1,32)_ = 2.102, p = 0.157; F_anisomycin(1,31)_ = 0.12, p = 0.731; F_cocaine × anisomycin_ = 0.564, p = 0.458; Two-way ANOVA stubby, F_cocaine(1,31)_ = 0.236, p = 0.63; F_anisomycin(1,31)_ = 0.167, p = 0.685; F_cocaine × anisomycin_ = 0.116, p = 0.735; Two-way ANOVA branched, F_cocaine(1,31)_ = 3.214, p = 0.082; F_anisomycin(1,31)_ = 0.188, p = 0.667; F_cocaine × anisomycin_ = 1.124, p = 0.297; Two-way ANOVA thin, F_cocaine(1,31)_ = 28.79, p<0.001; F_anisomycin(1,31)_ = 0.087, p = 0.769; F_cocaine × anisomycin_ = 0.101, p = 0.752). * p<0.05; ** p<0.01; *** p<0.001; n.s. (not significant) vs Saline + vehicle group; **N.S.** (not significant) vs Cocaine + vehicle group, Bonferroni post-hoc test, n = 7 to 10 animals/group.

Our data suggested that the increase of DeS in the NAcc, observed after cocaine-induced CPP, might be important for drug reward. So, we investigated the effect of an inhibition of DeS formation on cocaine-induced neuroplasticity and CPP. Blockade of new DeS formation was achieved by using anisomycin, a widely used protein synthesis inhibitor, known to inhibit growth of new DeS [27]. This compound was bilaterally infused in the NAcc core or shell after each drug conditioning sessions in CPP at a dose known to block protein synthesis (see Materials and Methods section). Immediately after the test, rats were perfused with fixative, their brains removed and fixed for subsequent DeS analysis. When injected in the NAcc core, anisomycin was able to block cocaine-induced CPP (Fig. 5A) and augmentation of DeS density in both NAcc core (Fig. 5B and 5C) and shell (Fig. 5D and 5E). However, infusion of anisomycin in the NAcc shell immediately after conditioning failed to block CPP (Fig. 6A) as well as increase of spines density in the NAcc core (Fig. 6B and 6C) or shell (Fig. 6D and 6E) promoted by cocaine. Interestingly, when anisomycin was injected in the shell 4 hours after conditioning, it blocked cocaine-induced CPP (Fig. 7A) and DeS increase in NAcc shell (Fig. 7D and 7E) but not in core (Fig. 7B and 7C). Conversely, anisomycin injection in the core 4 hours after conditioning did not inhibit CPP (Fig. 8A) or DeS density increase in NAcc core (Fig. 8B and 8C) or shell (Fig. 8D and 8E). Anisomycin alone was devoid of any effect on behavior and DeS density (Figs. 5, 6, 7 and 8).

## Discussion

A major challenge in drug addiction research is the understanding of brain mechanisms underlying drug-induced behaviors. Since cocaine was demonstrated to increase dendritic spine density in some brain regions [28], changes in brain structural plasticity induced by drugs of abuse have received a great attention. However, few studies have addressed the relationship between this structural plasticity and behavior associated to dependence. In most of these studies, different groups of animals and different drug treatments were used for the behavior and DeS quantitative analysis [11], [12], [29]. Moreover, withdrawal periods were frequently included between behavioral assessment and spine analysis [8]. As a consequence, it led to contradictory results regarding the role of DeS in cocaine rewarding effect. Thus, we designed a time- and subject-matched protocol in which DeS analysis were immediately realized after behavioral studies and on the same animals to demonstrate a role of DeS in cocaine rewarding effects.

**Figure 9:**
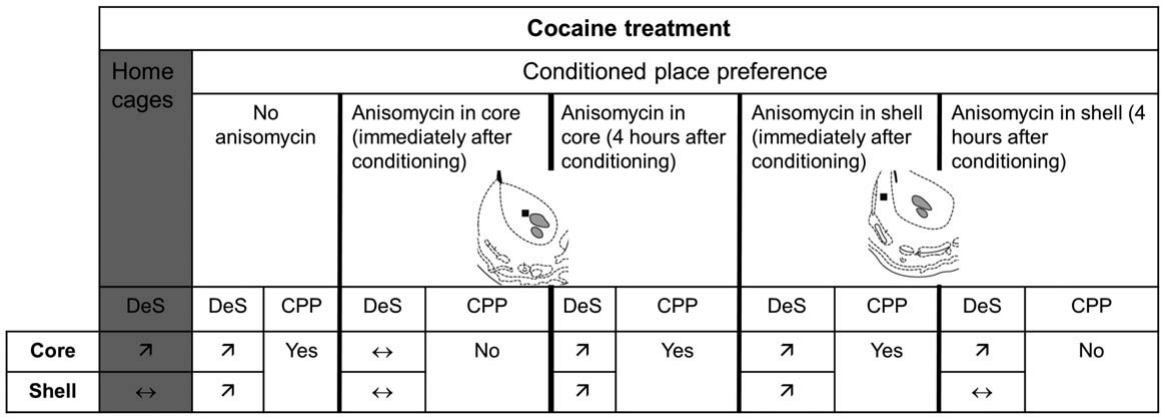
Cocaine effects on neuroplasticity and behavior. This figure presents the major findings in this study regarding relationship between structural plasticity induced in NAcc core and shell by cocaine and its behavioral effects measured by conditioned place preference (CPP). DeS, dendritic spines; ↗, increase; ↔, no change. Inset : partial coronal section from the atlas of Paxinos and Watson [14] showing NAcc core and shell; black squares represent the sites of injection in Nacc core (+1.7 mm from the bregma, ±1,8 mm lateral to the midline, and −6.3 mm under the skull surface) or shell (+1.2 mm from the bregma, ±0.8 mm lateral to the midline, and −6.7 mm under the skull surface); in grey : anterior commissure.

We quantified cocaine rewarding effects by using the CPP paradigm since this test allowed the measurement of a behavioral response in a drug-free state. Our data showed a correlation between CPP and increase in DeS density in the NAcc core and shell induced by cocaine (Fig. 9). This was consistent with the role of the NAcc in CPP [30]. In another animal model largely used in the addiction field, the self-administration paradigm, a DeS increase in both NAcc core and shell was also demonstrated. In this case, DeS were analyzed after one month withdrawal period [7]. However, our results showed that protracted withdrawal was not necessary to observe structural changes in brain. Same observation was made in ventral tegmental area where a DeS increase was detected 24 hours after an acute cocaine injection [31].

Although the role of the NAcc in neuroplasticity and reinforcing effects of cocaine is well established, the precise functions of its different subdivisions (core and shell) remain yet unclear. Our data showed that DeS density was increased only in the NAcc core after cocaine treatment in home cages (Fig. 9). As previously mentioned while several authors have emphasized the importance of a withdrawal period associated with cocaine-induced structural plasticity in the nucleus accumbens [7], [8], others recent studies have challenged this notion by demonstrating an increase of spine density 1 or 2 days after the final cocaine injection [32], [33], [34], [35]. Regarding the specific results published by Li et al. [8], they observed that cocaine treatment increased spine density in the nucleus accumbens shell (whatever the doses of cocaine used, 15 mg/kg and 30 mg/kg) and in the core (at the highest dose of cocaine, 30 mg/kg) in the “Home group”, two weeks after the last injection of cocaine. Some possible explanations for this disparity between this study and our study, are the differences in animal strains (rats from different vendors, Sprague-Dawley from Harlan vs Sprague-Dawley from Janvier) [36], differences in the doses of cocaine used (20 mg/kg in our study), or differences in the behavioral test used (sensitization to locomotor activity vs CPP).

Altogether, our data suggest that pharmacological effects of cocaine could be dissociated from its behavioral effects. In this case, the NAcc core and shell would be independent, with the shell involved in the learned associations between the effects of the drug and the environment while the core might be involved in pharmacological effects of cocaine. Our results are in good agreements with those of Sellings and co-corkers showing that medial shell lesions impair cocaine CPP acquisition, whereas core lesion has no effect [4]. Moreover microinjection of cocaine in the shell, not in the core, induces CPP [3].

However situation seems to be more complicated regarding our data obtained with the protein synthesis inhibitor, anisomycin. Indeed, we showed that blocking protein synthesis in the core inhibited cocaine-induced CPP and DeS increase in both NAcc core and shell (Fig. 5, 9). First, this suggests that the NAcc core plays a key role in cocaine-induced CPP. This is consistent with recent findings, where intra-core, not intra-shell, injection of a mu opioid receptor antagonist impaired the acquisition of cocaine-induced place preference [37]. Second, it suggests that cocaine-dependent neuroplasticity in the shell, occurring during CPP, depends on neuroplasticity changes in the core. This was confirmed by our results with anisomycin injection in the shell. When anisomycin was injected in the NAcc shell after conditioning, no effect on cocaine-induced CPP as well as neuroplasticity was observed (Fig. 9). Several hypotheses might explain these results. First, the mechanisms of DeS formation in the shell are not protein synthesis-dependent, which is very unlikely. Second, it is known that maximal inhibition of protein synthesis after anisomycin injection occurred within 3 hr postinjection [38], thus in our experimental conditions, protein synthesis blockade in the shell would occur too early. This suggests a time-dependent sequence of changes in neuroplasticity induced by cocaine starting with DeS increase in the core followed by neuroadaptations in the shell. Indeed, results obtained with anisomycin injected in the shell four hours after conditioning confirmed this second hypothesis. Indeed, in this case, we observed a blockade of DeS increase in shell and cocaine-induced CPP (Fig. 9). These results strongly suggested a transfer of neuroplasticity from core to shell, required for cocaine reward. This was confirmed by the lack of effect of anisomycin injection in the core four hours after conditioning. Core and shell are usually considered as two functionally independent subdivisions of nucleus accumbens, having distinct role in drug reward [3], [4], [37]. When interactions between core and shell have been described they were always from shell to core [39] probably via a shell-to-core loop [40]. Therefore, our data, supported by anatomical evidences of a direct connection from core to shell [41], represents a major breakdown in this concept of the nucleus accumbens functioning as they suggest that modifications in shell are dependent from those occurring in core.

Besides being a potent translational inhibitor, anisomycin was demonstrated to activate stress- dependent JNK (c-Jun N terminal kinases) and p38 MAPK (mitogen-activated protein kinase), and to weakly activate ERK (extracellular signal-regulated kinases) 1/2 pathway [42], [43], [44], [45], [46]. However, it is not the activation of ERK that is able to reduce the morphological changes induced by repeated cocaine administration, but rather its inhibition, as the selective ERK inhibitor, SL327 is able to block cocaine-induced increase in dendritic spine density of medium spiny neurons in the nucleus accumbens [35]. Thus, it is very unlikely that the effects observed in our study following anisomycin injection could be due to activation of MAPK signaling.

As they receive glutamatergic and dopaminergic inputs, DeS are thought to play a major role in neurobiological mechanisms of drugs of abuse. Indeed, glutamate has a major role in drug relapse [47] and dopamine is directly involved in drug reinforcing properties [48]. However, the precise function in behavior of DeS density variations after drug exposure remains unknown. Actually, conflicting results are found for cocaine since inhibition of DeS formation either favors [11], [49], [50] or blocks [12] cocaine-induced behavioral responses. Our data supported that blocking cocaine-induced DeS increase, with anisomycin, resulted in inhibition of CPP learning. Moreover, they could shed a new light on the numerous studies showing that blocking protein synthesis impaired learning processes. Indeed, it is the first time to our knowledge that blocking protein synthesis *in vivo* inhibited dendritic spine growth, since only *in vitro* data exist [27]. Thus, the effect of anisomycin on learning could be due to an inhibition of dendritic spine growth as these spines are found to be crucial in learning processes [51].

An important question regarding DeS concerns their functionality. Indeed, some studies have shown that after a cocaine treatment, a protracted withdrawal (at least 2 weeks) was necessary to induce functional changes in the NAcc. These functional changes would be due to an increase of AMPA receptor surface expression [52], [53] that would increase glutamatergic transmission in the NAcc [54]. Previous studies have demonstrated a positive correlation between spine size and AMPA current [55]. So, as we found an increase of stubby spines in the NAcc core of animals demonstrated a cocaine-induced CPP, one might hypothesize that these spines are functional as they would carry AMPA receptor. This data are consistent with those of Dobi an co-workers, who found that a short withdrawal period (2 days) was sufficient to induce synaptic adaption in NAcc core MSN [32].

In conclusion, we clearly demonstrated a correlation between cocaine rewarding effect and DeS increase in the NAcc core and shell. We also showed that when cocaine treatment was administered in home cages, DeS density was only increased in the NAcc core. Finally, blocking protein synthesis with anisomycin in the NAcc core, immediately after conditioning (but not after four fours), resulted in inhibition of cocaine-induced CPP and DeS increase in both core and shell. Whereas anisomycin injection immediately after conditioning in the NAcc shell had no effects on neuroplasticity or behavior, injection four hours after conditioning inhibited DeS increase in shell and cocaine-induced CPP. All these data demonstrated that a transfer of neuroplasticity from nucleus accumbens core to shell, was required for cocaine rewarding effects. This revealed a new model of nucleus accumbens functioning that should be taken into account when investigating behaviors involving this essential brain structure, such as decision making.
